# Distinct Functions and Assembly Mechanisms of Soil Abundant and Rare Bacterial Taxa Under Increasing Pyrene Stresses

**DOI:** 10.3389/fmicb.2021.689762

**Published:** 2021-07-02

**Authors:** Yuzhu Dong, Shanghua Wu, Ye Deng, Shijie Wang, Haonan Fan, Xianglong Li, Zhihui Bai, Xuliang Zhuang

**Affiliations:** ^1^Key Laboratory of Environmental Biotechnology, Research Center for Eco-Environmental Sciences, Chinese Academy of Sciences, Beijing, China; ^2^College of Resources and Environment, University of Chinese Academy of Sciences, Beijing, China

**Keywords:** assembly processes, abundant and rare taxa, biodegradation, environmental adaptability, polycyclic aromatic hydrocarbons

## Abstract

Elucidating the relative importance of species interactions and assembly mechanisms in regulating bacterial community structure and functions, especially the abundant and rare subcommunities, is crucial for understanding the influence of environmental disturbance in shaping ecological functions. However, little is known about how polycyclic aromatic hydrocarbon (PAH) stress alters the stability and functions of the abundant and rare taxa. Here, we performed soil microcosms with gradient pyrene stresses as a model ecosystem to explore the roles of community assembly in determining structures and functions of the abundant and rare subcommunities. The dose–effect of pyrene significantly altered compositions of abundant and rare subcommunities. With increasing pyrene stresses, diversity increased in abundant subcommunities, while it decreased in the rare. Importantly, the abundant taxa exhibited a much broader niche width and environmental adaptivity than the rare, contributing more to pyrene biodegradation, whereas rare taxa played a key role in improving subcommunity resistance to stress, potentially promoting community persistence and stability. Furthermore, subcommunity co-occurrence network analysis revealed that abundant taxa inclined to occupy the core and central position in adaptation to the pyrene stresses. Stochastic processes played key roles in the abundant subcommunity rather than the rare subcommunity. Overall, these findings extend our understanding of the ecological mechanisms and interactions of abundant and rare taxa in response to pollution stress, laying a leading theoretical basis that abundant taxa are core targets for biostimulation in soil remediation.

## Introduction

Polycyclic aromatic hydrocarbons (PAHs) have been extensively researched on account of their biotoxicity and high detection rate in the environment (Ma and Cao, 2010; Keith, 2015). Soil is the reservoir and transfer station of PAHs in the environment, which cause great effects on microorganisms and soil function (Crampon et al., 2018). The soil microbial community consists of a few taxa with high abundance (defined as abundant bacterial taxa) and a great quantity of taxa with low abundance (defined as rare bacterial taxa) (Hanson et al., 2012; Jousset et al., 2017), and prior studies indicated that abundant and rare microbial taxa often exhibited different distribution patterns and functional traits (Xue et al., 2020; Zhao et al., 2020). To date, some studies have evaluated the influence of PAHs on the whole bacterial community (Gao et al., 2019) and found obvious changes in community compositions and functions of soil microecosystems (Lors et al., 2010; Shahsavari et al., 2019). However, it is not clear how the abundant and rare bacterial taxa respond and adapt to different levels of PAH disturbance in soils and, in turn, the dose–effect of PAHs on the functions of the two subcommunities.

Recently, a growing body of research has emphasized the ecological importance of rare taxa (Lynch and Neufeld, 2015; Jousset et al., 2017), and distinct succession patterns and functional characteristics were found in abundant and rare taxa (Jia et al., 2018; Jiao and Lu, 2020b; Rocca et al., 2020). The abundant taxa are usually perceived as the most active category in biogeochemical cycles, especially carbohydrate metabolism, and take up the coring niche (Jiao et al., 2017; Kurm et al., 2019; Liang et al., 2020). Besides, due to their high abundance, the abundant taxa might present greater survivability to environmental stresses (Jiao et al., 2019; Jiao and Lu, 2020b). As for the rare taxa, according to numerous studies, they serve as a reservoir of genetic and functional diversity for the whole community and contribute to the maintenance of microbial diversity (Jousset et al., 2017; Rocca et al., 2020). That is, when faced with environmental disturbance, the rare taxa could respond promptly to maintain community stability (Ji et al., 2020; Zhang et al., 2020). For example, some rare taxa may turn to be dominant taxa in the community to enrich ecosystem functions for the disturbance (Jiao et al., 2019; Du et al., 2020). The interaction and transformation of abundant and rare bacterial taxa are critical to maintaining soil functional redundancy and community stability (Li et al., 2019; Liang et al., 2020). Exploring the co-occurrence relationship and succession pattern between abundant and rare bacterial taxa is conducive to identify the keystone taxa in soil microecosystems, so as to better predict and restore soil functions under different levels of PAH pollution (Stegen et al., 2012; Jiao and Lu, 2020a). But so far, very few works have addressed the mutual relations and succession patterns of the abundant and rare bacteria taxa under different levels of PAH concentrations.

Community assembly, studying the processes that shape the traits and abundance of taxa in ecological communities, is a key issue in evaluating the influence of environmental pollutants on the soil bacterial community and in turn affects the transfer and biodegradation of the pollutants (Stegen et al., 2013; Jia et al., 2018; Guittar et al., 2019). Since there are distinct ecological responses to environmental changes, the abundant and rare subcommunities might be dominated by different assembly processes under disparate disturbances (Jia et al., 2018; Liang et al., 2020). Some researchers suggested that abundant and rare bacterial taxa have a similar community assembly mechanism (Liao et al., 2017), while others reached the opposite conclusion that there is a considerable discrepancy in the proportion of stochastic and deterministic processes in the assembly of abundant and rare subcommunities (Du et al., 2020; Ji et al., 2020). Obviously, we still lack a comprehensive understanding of the universality in community assembly of abundant and rare bacterial communities under specific environmental disturbances. Clarifying the essential mechanisms for microbial succession and assembly under PAH stress has vital importance on soil microbial remediation (Dua et al., 2002; Gavrilescu et al., 2015).

In this study, we chose pyrene, four-aromatic ring model compounds for PAHs, to establish a soil microcosm incubation experiment in order to simulate different pollution levels with the purpose of: (i) clarifying the uncertain succession patterns (such as diversity, distribution, function) of abundant and rare bacterial taxa under different levels of pyrene stresses; (ii) revealing the co-occurrence relationships of the rare and abundant bacterial taxa in response to different levels of pyrene stresses; (iii) assessing the major processes controlling the assembly of the abundant and rare bacterial subcommunities along with pyrene stresses. Based on different ecological functions and environmental adaptations of abundant and rare bacterial taxa, we hypothesized that soil abundant bacterial taxa may have broader adaptivity to serious PAH stresses than rare bacterial taxa and distinct mechanisms dominated the assembly of the abundant and rare subcommunities. Our study could help predict the responses of soil bacteria to environmental pollutions and understand the generation and maintenance of bacterial functions in further *in situ* bioremediation technologies.

## Materials and Methods

### Soil Sampling and Experimental Setup

Surface soil samples were collected by thoroughly mixing several soil cores from a farmland (40°23′ E and 116°40′ N) in Changping District, Beijing, North China. All the samples were packed in sterile self-sealing bags and then sent to the laboratory immediately under dry ice conditions. The soil samples were passed through a 2-mm sieve after naturally air-dried under dark conditions to ensure homogeneity for subsequent experiments. The soil had a pH of 7.9, and the soil organic carbon and total nitrogen concentrations were 1.12 and 1.34 g kg^–1^, respectively. The total concentration of 16 Environmental Protection Agency (EPA) priority PAHs in this soil was 66 μg kg^–1^, which was far less than the standards of unpolluted soils (<200 μg kg^–1^) according to the classification of PAH pollution levels indicated by Maliszewska-Kordybach (1996). Besides, the soil also was below the soil pollution risk screening values for agricultural and development land from the Soil Environmental Quality Risk Control standard for soil contamination of agricultural land (GB15618-2018) and development land (GB36600-2018) issued by the Ministry of Ecology and Environment of China.

Five treatments were carried out with six replicates: unpolluted soil (Control Treatment) and polluted soils under four different pyrene concentrations (1, 10, 100, and 500 mg pyrene per kilogram dry soil, represented by PYR1, PYR10, PYR100, and PYR500, respectively). Pyrene-polluted soils were prepared in reference to the method of Brinch et al. (2002). Briefly, acetone stock solutions with different concentrations of pyrene were prepared first and then the same volume of stock solutions was spiked into 250-g homogenized soils. After the acetone completely evaporated, the spiked soils were thoroughly mixed with the remaining 750-g unpolluted soils. Meanwhile, an equal volume solution of pure acetone was added to the unpolluted soils in the same procedure to eliminate the effects of acetone.

For each treatment, 10-g pretreated soils were placed in a 120-ml serum bottle; then, sterile water was used to keep the soil moisture at about 60% every 2 days. All the microcosms were incubated for 35 days at 25°C in the dark. At the end of the 35-day incubation period, the pyrene concentration in Treatment PYR1 was lower than the detection limit, and all the soil CO_2_ emission rates showed a downward trend. Therefore, soil samples were stored at −80°C for microbial analyses.

Residual pyrene in soils was obtained by accelerated solvent extraction (ASE 350, Dionex, Thermo Scientific, United States) and solid-phase extraction purification and quantified by gas chromatography coupled to mass spectrometry (GC-MS, Shimadzu, Kyoto, Japan). The detailed parameters of GC-MS were 50°C hold for 2 min, rise to 180°C at a rate of 20°C/min, increase at 10°C/min to 290°C, then hold for 10 min (Rombolà et al., 2015).

To further confirm the results that pyrene stresses influenced the abundance of abundant and rare taxa, and the abundant taxa played an important role in pyrene degradation, unpolluted soils from four different places (Qingdao, Changsha, Kunming, and Guangzhou, represented by QD, CS, KM, and GZ, respectively) in China were collected to establish the microcosm. The PYR500 and its controls (soils without pyrene) were harvested after 35 days of cultivation and subjected to DNA extraction, 16S rRNA gene sequencing, and the further analysis.

### DNA Extraction and Illumina Sequencing

Extraction and purification of total bacterial genomic DNA from microcosms were performed using a FastDNA^®^ SPIN Kit for Soil (MP Biochemicals, United States) following the manufacturer’s instructions. The quantity and purity of soil DNA were determined by a Nanodrop 2000 UV-Vis Spectrophotometer (NanoDrop Technologies, Wilmington, DE, United States). DNA was quantified for *nidA* gene (pyrene dioxygenase gene) through the primer *nidA*-F (5′-TTC CCG AGT ACG AGG GAT AC-3′) and *nidA*-R (5′-TCA CGT TGA TGA ACG ACA AA-3′) (Ren et al., 2016). The V3–V4 region of the bacterial 16S rRNA gene was amplified using the primer 338F (5′-ACT CCT ACG GGA GGC AGC AG-3′) and 806R (5′-GGA CTA CHV GGG TWT CTA AT-3′) (Lee et al., 2017). The 20-μl PCR mixture contained 4 μl of 5 × FastPfu buffer, 2 μl of 2.5 mM dNTPs, 0.8 μl of 5 μM each primer (forward and reverse), 0.4 μl of TransStart Fastpfu DNA polymerase, 0.2 μl of bovine serum albumin, and 10 ng of sample DNA. The PCR reaction conditions were 95°C for 3 min, followed by 26 cycles of 30 s at 95°C, 55°C for 30 s, 72°C for 45 s, and a final extension at 72°C for 10 min. Each PCR product was sequenced on the Illumina MiSeq PE 300 × 2 sequencer at Majorbio Bio-Pharm Technology Co., Ltd. (Shanghai, China).

### Sequence Analysis

The Galaxy pipeline at the Research Center for Eco-Environmental Sciences, Chinese Academy of Sciences^1^, was used to process and analyze the high-throughput sequencing data (Feng et al., 2017). Briefly, paired-end reads were merged to a sequence of each sample according to the overlapped regions of reads (Zhang et al., 2017). Meanwhile, quality control and filtering were carried out to remove reads with lengths less than 50 bp, average score less than 20, and containing N bases. Effective sequences of each sample were obtained by distinguishing according to their unique barcodes and primer sequences with Fastp (Chen et al., 2018) and FLASH (Magoc and Salzberg, 2011). For the 30 soil samples, the mean average length was 418.87 ± 1.09 bp, and 1,374,419 quality-filtered clean sequences were obtained, ranging from 39,134 to 51,214 with a mean of 45,814 sequences per sample. Operational taxonomic units (OTUs) were picked using UPARSE at 97% similarity level, and sequences were then assigned to the SILVA reference using the RDP classifier (Quast et al., 2013). To compare different samples, we used a randomly selected subset of 39,134 sequences from each sample to normalize sequencing effort across samples. Finally, a resample OTU table was obtained for further statistical analysis.

### Data Analyses

Generally, different cutoffs of the relative abundance are applied to distinguish abundant and rare bacterial taxa. In our study, (i) OTUs with a relative abundance ≥ 1% in a sample were defined as abundant bacterial taxa, (ii) OTUs with a relative abundance < 0.01% in a treatment were defined as rare bacterial taxa, and (iii) OTUs with a relative abundance < 1% in a treatment and ≥ 0.01% in a sample were defined as moderate taxa (Ji et al., 2020; Liang et al., 2020; Mo et al., 2021).

Here, α-diversity indices (Shannon-Wiener and Simpson) were calculated through the “vegan” package, and one-way ANOVA was used to determine whether there were any statistically significant differences among treatments, followed by the *post hoc* Tukey honestly significant difference (HSD) test. In addition, β-diversity was measured based on Bray–Curtis dissimilarity using metaMDS in “vegan” package, and the non-metric multidimensional scaling (NMDS) was visualized using “ggplot2” package (Oksanen et al., 2013). Furthermore, analysis of similarities (ANOSIM) was performed to test whether there were significant differences in the soil bacterial community structures at different treatments. Resistance of abundant and rare taxa in polluted soils was calculated based on the Shannon–Wiener index compared to the Control (Liang et al., 2020).

Co-occurrence network was carried out through Molecular Ecological Network Analysis Pipeline (MENA^2^) based on the Random Matrix Theory (RMT) and Spearman correlation (Deng et al., 2012, 2016). According to the RMT-based modeling, a cutoff was chosen to construct the network, and then global network properties were calculated including individual nodes’ centrality, degree, betweenness, and clustering coefficient. Integrating all the results obtained from MENA, co-occurrence network was visualized by Gephi.

To predict the functional composition of the microbial community in the samples, the OTU abundance table was first standardized to remove the influence of the copy numbers of 16S marker gene in the species genome through Tax4Fun (Ashauer et al., 2015). Then, the corresponding relationship between SILVA classification and Kyoto Encyclopedia of Genes and Genomes (KEGG) database was established to predict microbial community function. Linear discriminant analysis Effect Size (LEfSe) was carried out to find the functions most likely to explain the differences among treatments (Segata et al., 2011).

Niche width was measured according to Levins’ coefficient (Levins, 1968):

(1) $$B_{i}=1/\sum_{j=1}^{r}P_{ij}^{2}$$

where B_*i*_ is the habitat niche width of OTU_*i*_, and P_*ij*_ is the proportion of OTU_*i*_ in the total OTUs within a given resource state j. The average number of all OTUs’ B was calculated to represent the niche width of the bacterial community.

Threshold indicator taxa analysis (Baker and King, 2010) was performed to analyze the threshold value of each abundant and rare bacterial taxa in response to variation of pollutant concentration. The z score of abundant and rare bacterial taxa was used to integrate taxon occurrence, abundance, and directivity.

In order to uncover the assembly mechanism of abundant and rare subcommunities, we performed the Sloan neutral community model (Sloan et al., 2010) and null model (Stegen et al., 2012) using the R scripts. The neutral community model was fitted by the nonlinear least-square fitting method, and the 95% confidence interval was predicted by the “Hmisc” package (Miller et al., 2016). In terms of the null model, Beta Taxon Index (βNTI) and Raup–Crick (RC_*Bray*_) were calculated to represent phylogenetic and taxonomic diversity (Stegen et al., 2013; Zhou and Ning, 2017). |βNTI| > 2 indicates the dominance of deterministic processes, while |βNTI| < 2 indicates the dominance of stochastic processes. |βNTI| < 2 and RC_*Bray*_ < −0.95 represent homogenizing dispersal. |βNTI| < 2 and RC_*Bray*_ > 0.95 represent dispersal limitation. |βNTI| < 2 and |RC_*Bray*_| < 0.95 represent “undominated” assembly (mainly consists of weak selection, weak dispersal, diversification, and/or drift). βNTI < −2 represents homogeneous selection. βNTI > 2 represents variable selection.

### Accession Numbers

All raw sequences of Illumina sequencing in this study have been submitted to the NCBI Sequence Read Archive (SRA) database, and the BioProject accession numbers for this research were PRJNA638003 and PRJNA728746.

## Results

### Relative Abundance and Taxonomic Compositions of Abundant and Rare Bacterial Taxa

**FIGURE 1:**
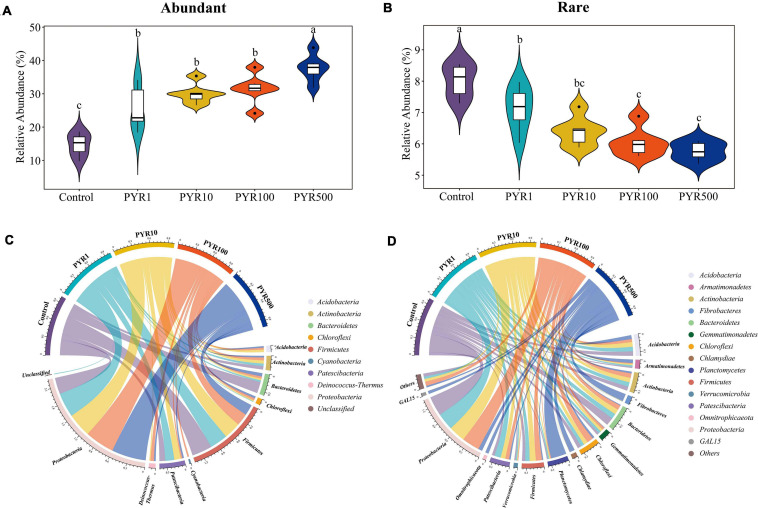
Relative abundance for abundant **(A)** and rare **(B)** bacterial taxa and taxonomic compositions for abundant **(C)** and rare **(D)** bacterial taxa. Each sample has six replicates, and the bar represents the standard deviation of the mean from the six replicates. Values assigned with the same letter were not significantly different by *post hoc* Tukey honestly significant difference (HSD) test (*p* ≤ 0.05).

In order to investigate the succession of abundant and rare bacterial taxa under different pyrene concentrations, we classified each OTU based on the selected cutoff and calculated the total relative abundance of abundant and rare bacterial taxa. Overall, abundant bacterial taxa (18–37 OTUs) accounted for only a small part of the bacterial community but represented 17.1%–43.8% of the soil microbial community abundance. However, 6,085–7,851 OTUs were attached to rare bacterial taxa, only accounting for 5.3%–8.5% of all sequences. With the increasing pyrene concentrations in soils, the proportion of abundant bacterial taxa increased, while the relative abundance of rare bacterial taxa decreased (Figures 1A,B; Supplementary Figure 1). Compared with polluted soils containing pyrene, the abundant bacterial taxa in unpolluted soil (Control) were significantly lower regardless of pyrene concentrations. In contrast, the rare bacterial taxa in the Treatment Control showed an opposite trend, significantly higher than polluted soils. The majority (94.6%–100%) of abundant bacterial taxa in polluted soils were from unpolluted soils, with about 21.6% from initially abundant bacterial taxa and 8.04% from initially rare bacterial taxa. However, a large part (63.3%–82.4%) of rare bacterial taxa in polluted soils was not detected in the original unpolluted soils (Supplementary Table 1).

To better figure out the microorganisms’ representative for abundant and rare bacterial taxa under different levels of pyrene stresses, we investigated the taxonomic compositions of the microbial community. The abundant bacterial taxa were comprised of nine phyla, dominated by Proteobacteria and Firmicutes, whereas up to 41 different phyla constituted the rare bacterial taxa (Figures 1C,D). Proteobacteria accounted for a high proportion in both abundant (28.5%–60.1%) and rare bacterial taxa (7.2%–35.6%). Interestingly, the relative abundance of Proteobacteria in abundant bacterial taxa increased with pyrene concentration, while a completely opposite trend was shown in rare bacterial taxa. As the pyrene concentration increased, the relative proportion of Firmicutes (19.1%–35.4%) in abundant bacterial taxa was on a downward path. Across abundant bacterial taxa, Chloroflexi and Deinococcus-Thermus were not involved in unpolluted soil but present in polluted soils (1.1%–3.8% and 2.0%–6.0% respectively), suggesting that the presence of pyrene activates specific bacteria taxa. Certain phyla occurred only in the rare bacterial taxa, such as Planctomycetes and Gemmatimonadetes. Compared with Control, the relative abundances of GAL15, Omnitrophicaeta, Planctomycetes, and Fibrobacteria in the rare subcommunity were significantly increased in PYR500. In particular, as for PYR500, the rare bacterial taxa were dominated by Planctomycetes (28.3%) and exhibited a higher relative abundance than other treatments (1.5%–2.4%).

### Diversities and Functions of Abundant and Rare Bacterial Taxa Under Different Pollution Concentrations

**FIGURE 2:**
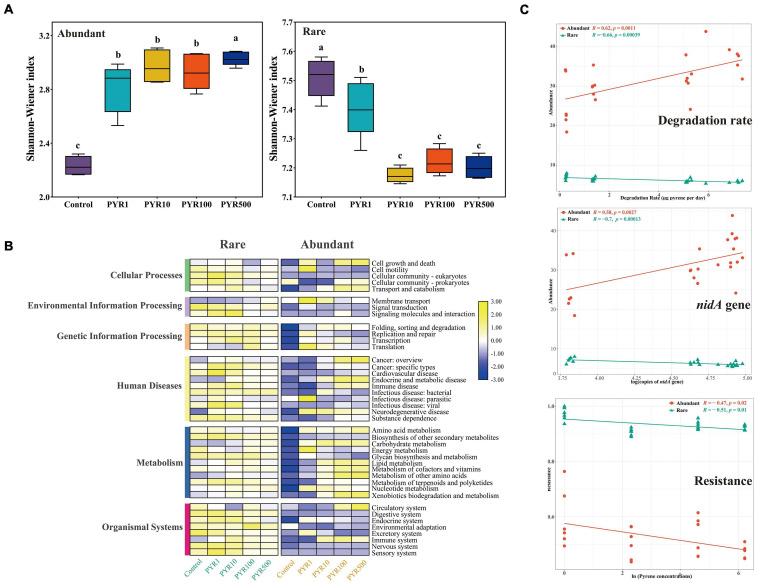
Changes of the diversities and functions in abundant and rare subcommunities under different levels of pyrene stress. **(A)** The Shannon–Wiener index and **(B)** heatmap of potential community functions based on Tax4Fun in abundant and rare subcommunities. **(C)** The correlation analysis between the abundance of abundant and rare taxa and pyrene degradation rate or logarithm of *nidA* gene copies; the correlation analysis between resistance of abundant and rare taxa and natural logarithm of pyrene stress concentrations.

Pollutants significantly changed the α-diversity of abundant and rare bacterial taxa (Figure 2A; Supplementary Figure 2). Compared with unpolluted soils, the Shannon–Wiener index of abundant bacterial taxa (2.16–3.96) in polluted soil increased, whereas rare bacterial taxa (7.07–7.58) decreased (Figure 2A; Supplementary Figure 3; ANOVA, *p* < 0.05). Moreover, we observed a significant influence of the pollution concentrations on both abundant and rare subcommunity structure based on Bray–Curtis dissimilarity (Supplementary Figure 4; ANOSIM, *R* = 0.899, *p* = 0.001 and *R* = 0.975, *p* = 0.001, respectively), and particularly, rare bacterial taxa were more sensitive than abundant bacterial taxa.

To further illustrate the differences between abundant and rare bacterial taxa under different pollution concentrations, community functions were analyzed by Tax4Fun based on KEGG Ortholog database. As for the six mainly functional categories, “Metabolism” always accounted for more than 60% across all samples in both abundant and rare bacterial taxa, followed by “genetic information processing” (17.4%–20.6%) (Supplementary Figure 5A). Rare bacterial taxa had five more functions than abundant bacterial taxa, mainly related to cellular processes (Supplementary Figure 5B). Interestingly, compared with Control, abundance of “Metabolism” showed an increasing trend along with increasing pollution concentrations no matter in abundant or rare bacterial taxa. However, there was a downturn in “genetic information processing” of polluted soils in both abundant and rare bacterial taxa (Figure 2B). Furthermore, based on LEfSe analysis, many functions significantly differed among treatments in both abundant and rare bacterial taxa with the LDA score (log 10) > 3.0 (Supplementary Figure 6). As for the abundant bacterial taxa, functions affiliated to the cellular processes were more abundant in unpolluted soils, while metabolism (related to degradation) showed a more important position in polluted soils. Moreover, functions related to bacterial membrane transport, such as ABC transporters, were more prevalent in the rare bacterial taxa under pyrene stresses.

In order to further verify the metabolism functions of abundant and rare bacterial taxa, correlation analysis between the abundance of abundant and rare taxa and pyrene degradation rates under different levels of pyrene stresses was performed (Figure 2C). Obviously, the degradation rate was significantly positively correlated with the abundance of abundant taxa (Spearman *R* = 0.62, *p* = 0.0011), whereas it was negatively correlated with the rare (*R* = −0.66, *p* = 0.00039). Interestingly, the same conclusion was found in the correlation between the copies of *nidA* gene (pyrene dioxygenase gene) and the abundance of abundant (Spearman *R* = 0.58, *p* = 0.0027) and rare (Spearman *R* = −0.7, *p* = 0.00013) taxa. When focusing on the resistance ability of subcommunities under stress environments, we found that the resistance of rare subcommunity (0.8886–0.9995) was much higher than that of abundant subcommunity (0.4481–0.7638), and both were negatively correlated with pyrene stress concentrations (rare: Spearman *R* = −0.51, *p* = 0.01; abundant: Spearman *R* = −0.47, *p* = 0.02).

### Co-occurrence Patterns of Abundant and Rare Subcommunities

To better examine the interaction between microorganisms with different abundances in the community, a subcommunity co-occurrence network was constructed at the OTU level based on the Spearman’s correlation relationships. The co-occurrence network exhibited a scale-free character (Supplementary Figure 7, R square power-law: 0.917), indicating a nonrandom structure. In the whole network, there were about 14, 79, and 290 OTU nodes of abundant, moderate, and rare bacterial taxa, respectively. Most abundant nodes tended to have edges with rare nodes. Two important node-level topological features of different subcommunities including degree and betweenness were performed to further resolve the differences. The values of node degree and betweenness were significantly higher for rare and moderate taxa than abundant bacterial taxa (Tukey HSD, *p* < 0.001 and *p* < 0.01 respectively), but there was no significant difference between rare and moderate taxa in both topological features (Tukey HSD, *p* > 0.05).

**FIGURE 3:**
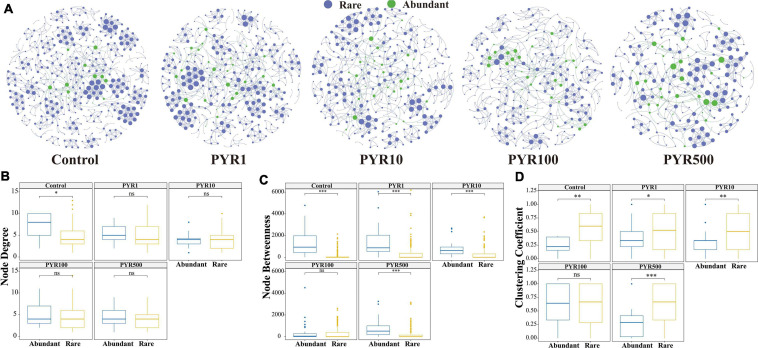
Network analysis **(A)** and topological relations **(B–D)** between abundant and rare taxa under different levels of pyrene stress. The blue and green nodes represent abundant and rare operational taxonomic units (OTUs), respectively. All the networks were visualized by Gephi. The size of each node is proportional to its number of connections. Node degree **(B)**, betweenness **(C)**, and clustering coefficient **(D)** of abundant and rare networks under different levels of polycyclic aromatic hydrocarbon (PAH) stress. Asterisks indicate significance: ^∗^*p*-value < 0.05, ^∗∗^*p*-value < 0.01, ^∗∗∗^*p*-value < 0.001 based on Tukey honestly significant difference (HSD) test.

In order to further reveal the direct relationship between abundant and rare bacterial taxa, co-occurrence networks were built through linking abundant OTUs to rare OTUs under different pollution concentrations. With the increase of pollutant concentrations, the co-occurrence networks tended to be simplified, among which nodes and links showed a decreasing trend gradually (Figure 3). In particular, more nodes related to abundant bacterial taxa showed high levels in PYR500 compared with rare bacterial taxa. We also calculated three important node-level topological features of different subcommunities including degree, betweenness, and clustering coefficient (Figure 3). Importantly, we only found that the node degree of abundant bacterial taxa was significantly higher than that of rare bacterial taxa in unpolluted soils. Besides PYR100, other treatments of betweenness in abundant bacterial taxa were significantly higher than those in rare bacterial taxa (*p* < 0.05), and nodes with high betweenness had more control over the network, indicating that more information may be passed through abundant bacterial taxa. However, except PYR100, the clustering coefficient in abundant bacterial taxa was significantly lower than that in rare, suggesting that the adjacency points of rare bacterial taxa had a higher interconnection degree.

### Ecological Assembly Processes of the Abundant and Rare Subcommunities

**FIGURE 4:**
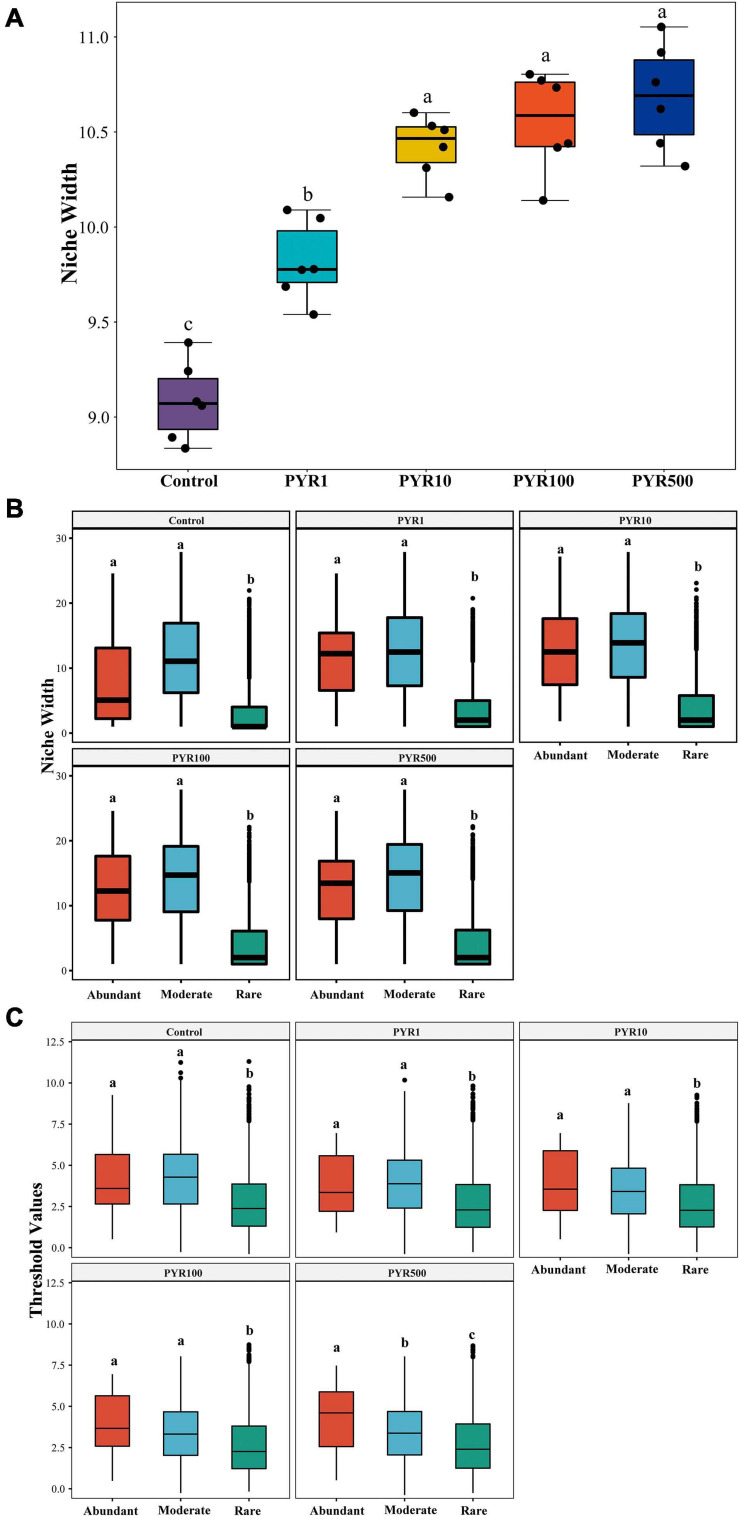
The environmental adaptability of the entire bacterial community **(A)** and subcommunities **(B,C)** under different levels of pyrene stress. The niche width of the **(A)** entire community and **(B)** subcommunities, **(C)** the environmental threshold to pyrene stresses of abundant and rare subcommunities. Values assigned with the same letter were not significantly different by *post hoc* Tukey honestly significant difference (HSD) test (*p* ≤ 0.05).

To examine the adaptive capacity of microorganisms in specific PAH-polluted environments, we calculated the average niche width of the community. Notably, along the increasing PAH concentrations in soil, the average niche width (8.83–11.05) of the community showed a trend of gradual increase (Figure 4A; ANOVA, *p* < 0.05). When the pyrene concentrations exceeded 10 mg kg^−1^ soils, the bacterial community had a significantly broader niche width than Control and PYR1 (Tukey HSD, *p* < 0.05). In addition, we performed the niche width of subcommunities to further refine the response of abundant and rare bacterial taxa to pyrene stresses. There was a significant difference across the niche width of three subcommunities in all treatments, and the rare subcommunity stayed at the narrowest niche width (Figure 4B). Interestingly, the niche width of abundant subcommunity was significantly broader than that of the rare subcommunity (Tukey HSD, *p* < 0.05), whereas it was narrower than that of the moderate subcommunity (Tukey HSD, *p* > 0.05). Moreover, we also discovered the consistent trend for the threshold values of subcommunities to respond to pyrene stresses in soils using threshold indicator taxa analysis (TITAN2), which is calculated based on the sum *z* scores for each taxon in subcommunities (Figure 4C). That is, the abundant subcommunity exhibited a significantly broader range of threshold for pyrene stresses than the rare subcommunity (Tukey HSD, *p* < 0.05).

**FIGURE 5:**
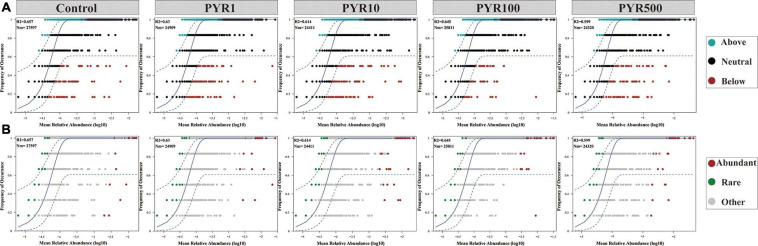
Neutral model (relative abundance–frequency relationships) of the entire bacterial community **(A)** and the abundant and rare taxa **(B)** under different levels of pyrene stresses. The dashed blue line represents the 95% confidence interval above and below the prediction (the solid blue line). R2 indicates the coefficient of the neutral fit, and Nm indicates the metacommunity size times immigration.

To address the effect of pyrene stresses on community and subcommunity assembly, we performed Sloan neutral community model to determine the relative importance of the neutral processes. Along the increasing pyrene concentrations, the goodness of fit (*R*^2^ = 0.599–0.657) of the neutral community model displayed a downward trend (Figure 5). We discovered that the neutral community model accounted for a large part of the community variance (84.4%–87.6%) between the occurrence frequency of OTUs and their mean relative abundance. There was a gradually decreasing trend in the Nm-value with the increased levels of PAHs pollution (Nm = 27,597–24,320). Almost all of the abundant bacterial taxa were distributed in the 95% confidence interval of the predicted neutral community model, whereas majority of the rare bacterial taxa were above the predicted occurrence frequency.

**FIGURE 6:**
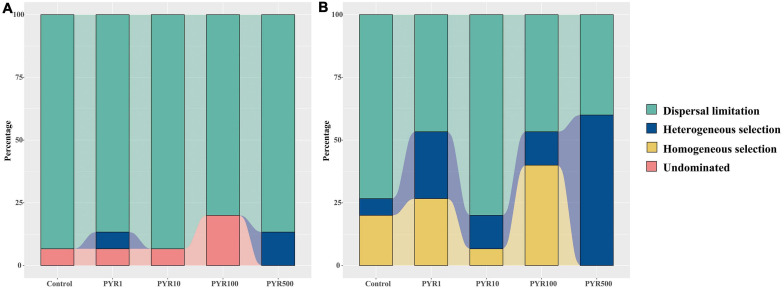
The fraction of assembly mechanism in abundant **(A)** and rare **(B)** subcommunities based on the null model.

To better illustrate the differences of assembly processes between the abundant and rare subcommunities, the null model was performed based on the βNTI. For the entire community, the βNTI values in Control were mainly between −2 and 2 but gradually below −2 along the pyrene concentrations (Supplementary Figure 9A). We found that the maximum mean βNTI values for abundant and rare subcommunities were both in PYR500 (Supplementary Figure 8). Majority of the βNTI values for the abundant subcommunity dominated between −2 and +2 in all treatments, whereas the distributions of βNTI gradually shifted with increasing pyrene concentration in the rare subcommunity from stochastic community assembly (−2 < βNTI < +2) to deterministic community assembly (|βNTI| > 2). Given the Raup–Crick distance based on taxonomic dissimilarity index, along the pyrene concentration, the stochastic assembly (dispersal limitation, 86.7%–93.3%) occupied a large proportion in the abundant subcommunity (Figure 6). However, the trend in the fraction of dispersal limitation was a decrease with pyrene concentration (from 73.3% at Control to 40% at PYR500) in the rare subcommunity. Importantly, in polluted soils (except PYR10), deterministic community assembly (heterogeneous selection and homogeneous selection) contributed more variation than stochastic community assembly (dispersal limitation). Similarly, the proportion of deterministic community assembly (heterogeneous selection and homogeneous selection) increased with the pyrene concentrations (Supplementary Figure 9B).

### Changes of Different Soils in Abundant and Rare Taxa Under Pyrene Stresses

To get more evidence, we performed microcosm incubation using four different soils and analyzed their abundant and rare taxa. Similarly, the relative abundance of abundant taxa in PYR500 (28.8%–76.7%) were significantly higher than those in Control (10.1%–49.2%) across all soil samples (*p* < 0.001). The rare taxa in PYR500 showed a significantly lower relative abundance than in Control (Supplementary Figure 10A; *p* < 0.01). Importantly, the pyrene dioxygenase gene copies (*nidA*) were also significantly positively correlated with abundance of abundant taxa (Supplementary Figure 10B; Spearman *R* = 0.69, *p* = 0.013), further suggesting that the abundant taxa played an important role in pyrene degradation. Moreover, the rare taxa presented a significantly higher resistance index compared with the abundant taxa (Supplementary Figure 10C; *p* < 0.001), implying that the rare taxa are critical for maintaining community stability.

## Discussion

**FIGURE 7:**
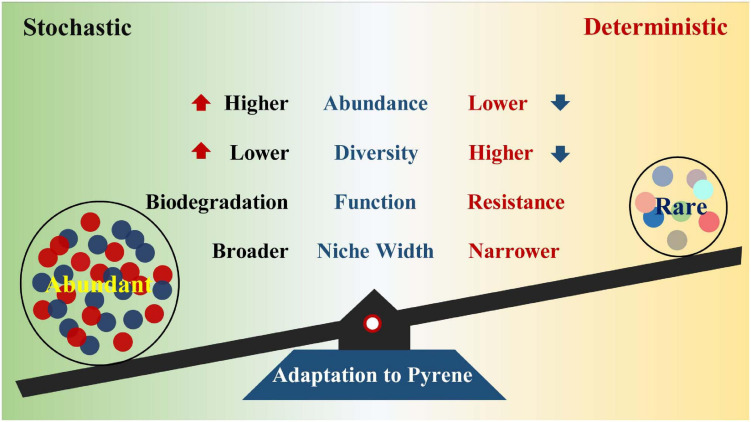
Functions of the abundant and rare bacterial taxa to adapt to pyrene stresses.

Previous studies have revealed the impact of PAHs on microbial communities (Lors et al., 2010; Niepceron et al., 2013; Crampon et al., 2018; Li et al., 2020), but few have reported the succession patterns and community assembly mechanisms of abundant and rare bacterial subcommunities under various pyrene stresses. In this study, microcosm was constructed to explore the strategies of soil abundant and rare bacterial taxa in response to diverse pyrene concentrations. Pyrene stresses increased the proportion and diversity of abundant bacterial taxa, whereas rare bacterial taxa presented an opposite trend. Compared with the rare bacterial taxa, abundant bacterial taxa had better adaptive capacity and broader niche width to PAH-polluted environment. The abundant taxa are more likely to degrade pollutants, while the rare taxa contribute more to community resistance. We also reveal that distinct community assembly processes drove the abundant and rare subcommunities under different levels of pyrene stresses (Figure 7).

### Broader Environmental Adaptations of Abundant Bacterial Taxa in Response to Pyrene Stresses

Increasing evidence has demonstrated the appreciable impact of PAHs on soil ecological functions (Wang et al., 2017; Liu et al., 2020). Numerous studies focused on the effects of PAH pollution in the natural environment on microbial community, but the dose–effects of pollutants are easily overlooked. Besides, although it has been well accepted that soils contaminated by PAHs have distinctive bacterial diversity and taxonomic compositions (Crampon et al., 2018; Ahmad et al., 2019), much less attention has been paid to the succession patterns (such as diversity, distribution, function) of abundant and rare bacterial taxa under different levels of pyrene stresses, not to mention their environmental adaptation.

Here in our study, results showed that there were markedly different succession patterns between the abundant and rare bacterial taxa under different levels of pyrene stresses. In terms of α diversity, the Shannon–Wiener index of rare bacterial taxa decreased with the increased pyrene concentrations, while the abundant bacterial taxa presented an opposite trend (Figure 2A; Supplementary Figure 3). Interestingly, the α diversity of the rare bacterial taxa was higher than the abundant bacterial taxa in all treatments (Supplementary Figures 2, 3), suggesting that the rare bacterial taxa made more contributions to the composition of overall community (Xue et al., 2018; Du et al., 2020). Due to their high diversity, the rare bacterial taxa could increase the functional redundancy of the community, further providing wider ecological buffering space to strive against a changing environment (Hausmann et al., 2016; Jiao et al., 2017; Kurm et al., 2019).

As to the distribution, the abundant and rare bacterial taxa significantly separated along the pyrene concentration gradients, the abundant bacterial taxa had a gradual increase in the proportion (Figure 1). The new abundant bacterial taxa in polluted soils mainly came from the rare and moderate bacterial taxa in unpolluted soil, primarily belonging to Alphaproteobacteria, Gammaproteobacteria (Proteobacteria), and Bacilli (Firmicutes) (Supplementary Table 1). A wave of recent studies suggested that the rare bacterial taxa could act as the “seed bank,” which plays an insurance effect on the whole community (Rocca et al., 2020). Our results supported prior studies reporting that the rare bacterial taxa have an opportunity to be activated to maintain the stability of the bacterial community under pollution stresses (Jousset et al., 2017). Intriguingly, four OTUs (OTU_2, OTU_27546, OTU_3, and OTU_8) belong to the rare species in uncontaminated soil before and turned into abundant under pyrene stresses (Supplementary Table 1). OTU_2 and OTU_27546 are aligned to Burkholderiaceae, while OTU_3 and OTU_8 belong to Paenibacillaceae and Bacillaceae, which all are known as typical PAH-degrading bacteria and carried several diverse ring-cleaving dioxygenase genes (e.g., 1,2 dioxygenase and 3,4-dioxygenase) (Ghosal et al., 2016; Li et al., 2018; Morya et al., 2020). However, most of the rare bacterial taxa in polluted soils were absent from the original unpolluted soil and have some unique phyla, such as Planctomycetes and Gemmatimonadetes, indicating that the rare bacterial taxa have environmental specificity under PAH selection (Kurm et al., 2019).

Historically, several studies have shown the occurrence of microbial adaptation in the environment after exposure to a xenobiotic chemical (Itrich et al., 2015; Poursat et al., 2019). PAH stresses altered the dynamic balance of abundant and rare bacterial subcommunities, further influencing environmental adaptation (Xue et al., 2018; Jiao and Lu, 2020b). Here, we discovered that the abundant bacterial taxa have broader adaptability to pollution stresses from two different aspects. First, the abundant bacterial taxa possess broader niche width than the rare, which was consistent with previous research (Figure 4B; Du et al., 2020; Jiao and Lu, 2020a). The niche width is an index of biodiversity of biological utilization resources, which means that the abundant bacterial taxa have the ability to make efficient use of various resources and survive in a more diverse environment compared with the rare bacterial taxa (Godoy et al., 2018). A prior study has demonstrated that many relatively abundant soil bacterial phylotypes could be found across a wide range of soils, suggesting the stronger environmental adaptability of abundant bacterial taxa (Delgado-Baquerizo et al., 2018). Moreover, the abundant bacterial taxa presented a higher response threshold to pyrene stresses, which proved that they can have a relatively durable survivability in polluted environment from another perspective (Figure 4C; Baker and King, 2010). Second, according to the correlation-based network analysis, we found that majority of the abundant bacterial taxa tend to be connected with the rare bacterial taxa, inferring that the two subcommunities were simultaneously affected by pyrene stresses (Figure 3). This is also demonstrated by the simplification of the network structures between abundant and rare bacterial taxa along the pyrene gradient. The topology of network could reflect the interaction among taxa, for example, node degree can indicate the links, and the node betweenness represents the impact on connected nodes (Feng et al., 2017). Our results showed that the node degree and betweenness of the abundant subcommunity were higher than those of the rare subcommunity, indicating that the abundant bacterial taxa were inclined to occupy the core and central position in adaptation to the PAH stresses (Du et al., 2020).

Previous studies have shown that microorganisms can adapt to the environment by regulating their functions (Roller et al., 2013; Tikariha and Purohit, 2019). Based on the predicted subcommunity functions, the abundant bacterial taxa showed gradually enhanced metabolic functions along the PAH gradient and higher than that of the rare bacterial taxa. Importantly, functions related to xenobiotics biodegradation and metabolism (such as PAHs) and metabolism-specific simple carbohydrates (such as lipid, amino acids, and vitamins) presented higher relative abundance in the abundant subcommunities than the rare subcommunities (Figure 2B). This is similar to a previous work that concluded that high abundance taxa in polychlorinated biphenyl (PCB)-contaminated soils were relevant to PCB degradation (Xu et al., 2020). The enhanced xenobiotics metabolic capacity in abundant bacterial taxa was conducive to survival in different levels of pyrene stresses. Both pyrene degradation rate and *nidA* gene copies (pyrene dioxygenase gene) were significantly positively correlated with abundance of abundant taxa, negatively relative to the rare (Figure 2C), further proving abundant taxa rather than the rare taxa played an important role in PAH degradation. On the other hand, functions affiliated with membrane transport, particularly the ABC transporters, which are important for tolerance to many different kinds of pollutants, were more prevalent in the rare communities (Figure 2B). Meanwhile, the rare subcommunity displayed higher resistance than the abundant under different levels of pyrene stresses (Figure 2C). Together, these results indicated that the abundant bacterial taxa may play an important role in the degradation of pollutants, while the rare bacterial taxa play a key role in improving community tolerance (Jiao et al., 2017).

### Distinct Assembly Mechanisms of Abundant and Rare Subcommunities Under Pyrene Stresses

The assembly process of microbial community can inevitably affect the diversity and composition of soil microbiome, thus influencing the functions of soil microecosystem (Leibold et al., 2017). Therefore, a deeper knowledge of assembly mechanisms of abundant and rare subcommunities under pyrene stresses will lead to a better understanding of the adaptability of bacterial abundance to environmental disturbances. The neutral community model is a prediction model based on neutral theory, which is an effective method to infer whether the stochastic process is dominant in the community assembly (Sloan et al., 2010). Our results clearly showed that almost all the abundant bacterial taxa were present in the predicted neutral region, while most of the rare bacterial taxa were above the predicted neutral region (Figure 5). The neutral community model could not explain 100% of the variation in the microbial community, suggesting that there may be other community assembly mechanisms that lead to non-neutral distribution (Dini-Andreote et al., 2015; Hou et al., 2020). We further performed the null model to elucidate the assembly mechanism of abundant and rare subcommunities (Stegen et al., 2013; Wang et al., 2021). In terms of unpolluted soils, stochastic processes (mainly dispersal limitation, more than 70% and 90%, respectively) participated in shaping both abundant and rare subcommunity assemblies (Figure 6). As for the abundant subcommunity, dispersal limitation was always dominant, independent of the PAH concentrations. However, the prominent role of deterministic processes in shaping the rare subcommunity assembly emerged along the pyrene stresses, except PYR10. As expected, due to their essentially low relative abundance and narrow niche width, the rare bacterial taxa are more sensitive to environmental filtering and less competitive than the abundant bacterial taxa (Lynch and Neufeld, 2015; Liang et al., 2020; Rocca et al., 2020). Importantly, the abundant bacterial taxa with a broader niche width are likely to make competitive use of various resources and adapt well to specific ecosystems through active growth and high abundance (Li et al., 2019; Jiao and Lu, 2020a). This indicates that PAH-induced taxa assembly has a great influence on the composition of bacterial subcommunities. As for the stochastic processes dominated in rare PYR10, we consider it as the turning point of steady state, with uncertainty, because the Shannon–Wiener index drops off a cliff in rare bacterial taxa of PYR10, whereas there was no similar phenomenon in abundant bacterial taxa. Considering the results of the neutral model and null model, we revealed that the stochastic process and deterministic process, respectively, dominate the assembly of abundant and rare bacterial subcommunities in PAH-polluted soils.

In summary, our study provides a better understanding of succession patterns and subcommunity assembly processes underlying the abundant and rare bacterial taxa under different levels of pyrene stresses and reveals the importance of the abundant bacterial taxa on the maintenance of community stability and adaptation to harsh environments. The abundance, diversity, and metabolism of specific carbohydrates in the abundant bacterial taxa rose across the increasing pyrene concentrations. Higher abundance and broader niche width are beneficial for the abundant bacterial taxa to cope with pyrene stresses. The rare bacterial taxa with higher phylogenetic diversity serve as a “seed bank” and play a crucial role in improving community stress resistance. Stochastic processes were dominant in driving the assembly of the abundant subcommunity, whereas the relative importance of deterministic processes progressively increased with pyrene stresses. The results based on the abundant and rare bacterial taxa may conduce to broaden our horizon about understanding the assembly and maintenance of bacterial diversity and function responses to pollution stresses.

## Data Availability Statement

The datasets presented in this study can be found in online repositories. The names of the repository/repositories and accession number(s) can be found in the article/Supplementary material.

## Author Contributions

SWu designed the research. XZ, SWu, and ZB supervised the project. YDo, HF, XL, and SWa refined the experimental method. YDo performed the research and conducted the data analyses with assistance from SWu and YDe. YDo drafted the manuscript. XZ, SWu, and YDe reviewed and edited the manuscript. All authors contributed to the article and approved the submitted version.

## Conflict of Interest

The authors declare that the research was conducted in the absence of any commercial or financial relationships that could be construed as a potential conflict of interest.

## Footnotes

1 http://mem.rcees.ac.cn:8080/

2 http://ieg4.rccc.ou.edu/mena

## Supplementary Material

Supplementary Figures 1–10 Correlation between natural logarithm of pyrene concentrations and richness of abundant and rare bacterial taxa. Simpson index of abundant and rare bacterial taxa under different levels of pyrene stresses. Correlation between natural logarithm of pyrene concentrations and Shannon-Wiener index of abundant and rare bacterial taxa. nMDS was calculated based on Bray-Curtis dissimilarity and 95% confidence ellipse was added for each treatment. Comparison of potential functions in abundant and rare subcommunities among treatments based on Tax4Fun. The linear discriminant analysis (LDA) value distribution histogram of functions in abundant and rare subcommunities. Network and topological relation in the whole communities. NTI of abundant and rare bacterial taxa under different levels of pyrene stresses based on the null model. The NTI value and fraction of assembly mechanism in entire community based on the null model. Changes of different soils in abundant and rare taxa under pyrene stresses.

Supplementary Table 1 Information of abundant OTUs in treatments.
