# Supplementary material for: Distinct Functions and Assembly Mechanisms of Soil Abundant and Rare Bacterial Taxa Under Increasing Pyrene Stresses
Source: Front Microbiol. 2021 Jul 2;12:689762. doi: 10.3389/fmicb.2021.689762 (PMC8283415; doi:10.3389/fmicb.2021.689762)
Supplement: Supplementary Figures 1–10 — Correlation between natural logarithm of pyrene concentrations and richness of abundant and rare bacterial taxa. Simpson index of abundant and rare bacterial taxa under different levels of pyrene stresses. Correlation between natural logarithm of pyrene concentrations and Shannon-Wiener index of abundant and rare bacterial taxa. nMDS was calculated based on Bray-Curtis dissimilarity and 95% confidence ellipse was added for each treatment. Comparison of potential functions in abundant and rare subcommunities among treatments based on Tax4Fun. The linear discriminant analysis (LDA) value distribution histogram of functions in abundant and rare subcommunities. Network and topological relation in the whole communities. NTI of abundant and rare bacterial taxa under different levels of pyrene stresses based on the null model. The NTI value and fraction of assembly mechanism in entire community based on the null model. Changes of different soils in abundant and rare taxa under pyrene stresses. [file Data_Sheet_1.pdf]

Supporting Information

Distinct functions and assembly mechanisms of soil abundant and rare bacterial taxa under increasing pyrene stresses

Yuzhu Dong[1,2,†], Shanghua Wu[1,2,†], Ye Deng[1,2], Shijie Wang[1,2], Haonan Fan[1,2], Xianglong Li[1,2], Zhihui Bai[1,2] & Xuliang Zhuang[1,2,*]

[1] Key Laboratory of Environmental Biotechnology, Research Center for Eco-Environmental Sciences, Chinese Academy of Sciences, Beijing 100085, China

[2] College of Resources and Environment, University of Chinese Academy of Sciences, Beijing 100049, China

[†] Y. D. and S. W. contributed equally to this paper.

[*] **Corresponding author:** Xuliang Zhuang, E-mail address: xlzhuang@rcees.ac.cn

List of supporting materials:

1. Page 15, (including cover)
2. One table, Table S1
3. Eight Figures, Figure S1, S2, S3, S4, S5, S6, S7, S8, S9 and S10

Contents

Supporting Information 1

Figure S1 Correlation between natural logarithm of pyrene concentrations and richness of abundant and rare bacterial taxa 3

Figure S2 Simpson index of abundant (A) and rare (B) bacterial taxa under different levels of pyrene stresses. 4

Figure S3 Correlation between natural logarithm of pyrene concentrations and Shannon-Wiener index of abundant and rare bacterial taxa 5

Figure S4 nMDS was calculated based on Bray-Curtis dissimilarity and 95% confidence ellipse was added for each treatment. 6

Figure S5 Comparison of potential functions in abundant and rare subcommunities among treatments based on Tax4Fun. 7

Figure S6 The linear discriminant analysis (LDA) value distribution histogram of functions in abundant (A) and rare (B) subcommunities. 8

Figure S7 (A) Network in the whole communities. Node degree (B) and betweenness (C) of the network. 9

Figure S8 NTI of abundant and rare bacterial taxa under different levels of pyrene stresses based on the null module. 10

Figure S9 The NTI value (A) and fraction (B) of assembly mechanism in entire community based on the null model. 10

Figure S10 Changes of different soils in abundant and rare taxa under pyrene stresses. 11

Table S1 Information of abundant OTUs in treatments 12

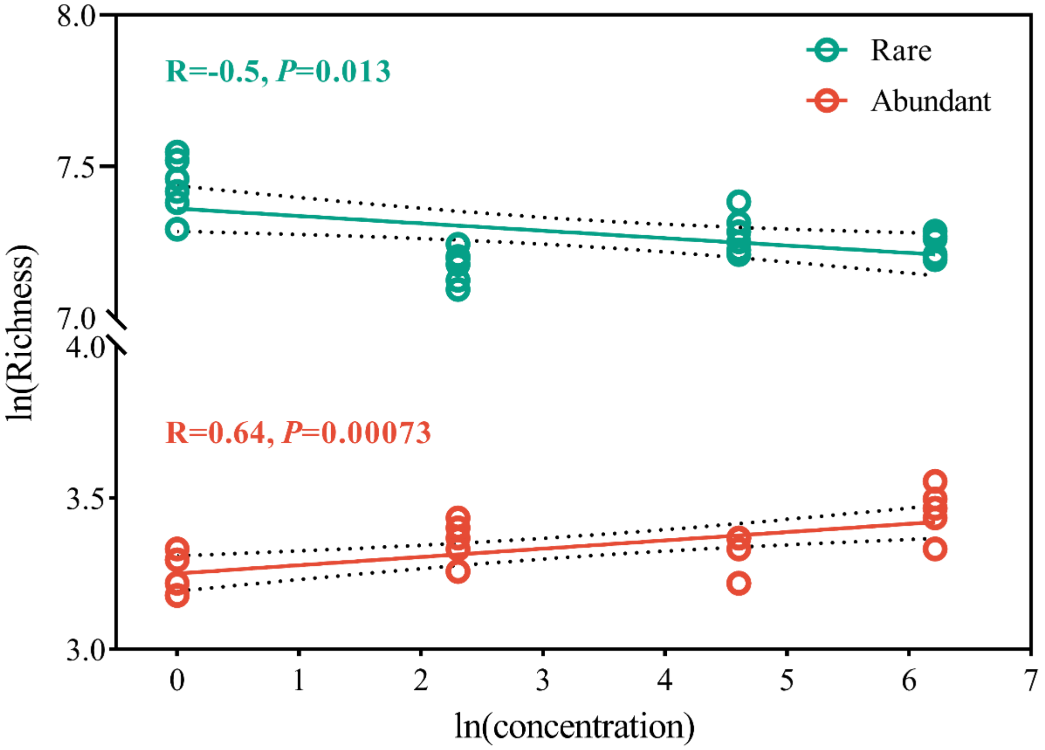

Figure S1 Correlation between natural logarithm of pyrene concentrations and richness of abundant and rare bacterial taxa

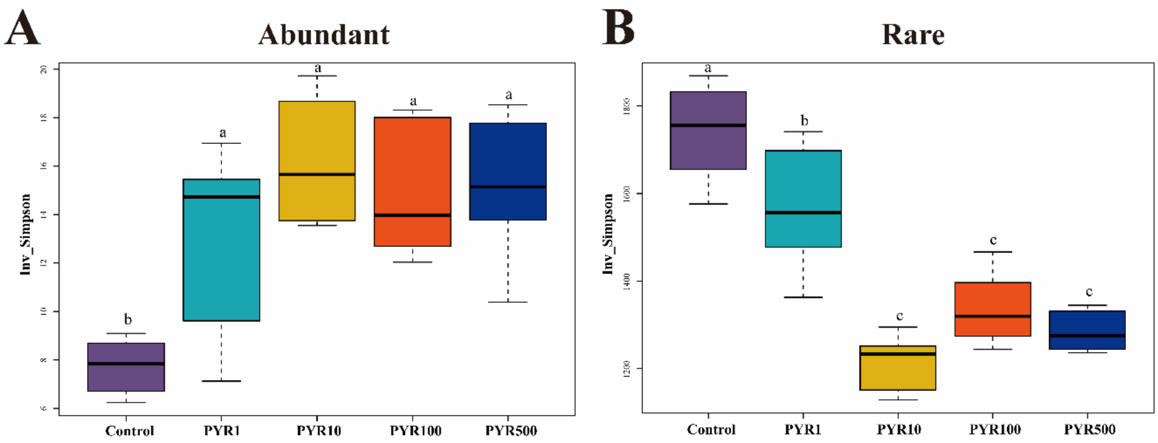

Figure S2 Simpson index of abundant (A) and rare (B) bacterial taxa under different levels of pyrene stresses. Each sample has 6 replicates, and the bar represents the standard deviation of the mean from the 6 replicates. Values assigned with the same letter were not significantly different by Tukey HSD post hoc ($P \leqslant 0.05$)

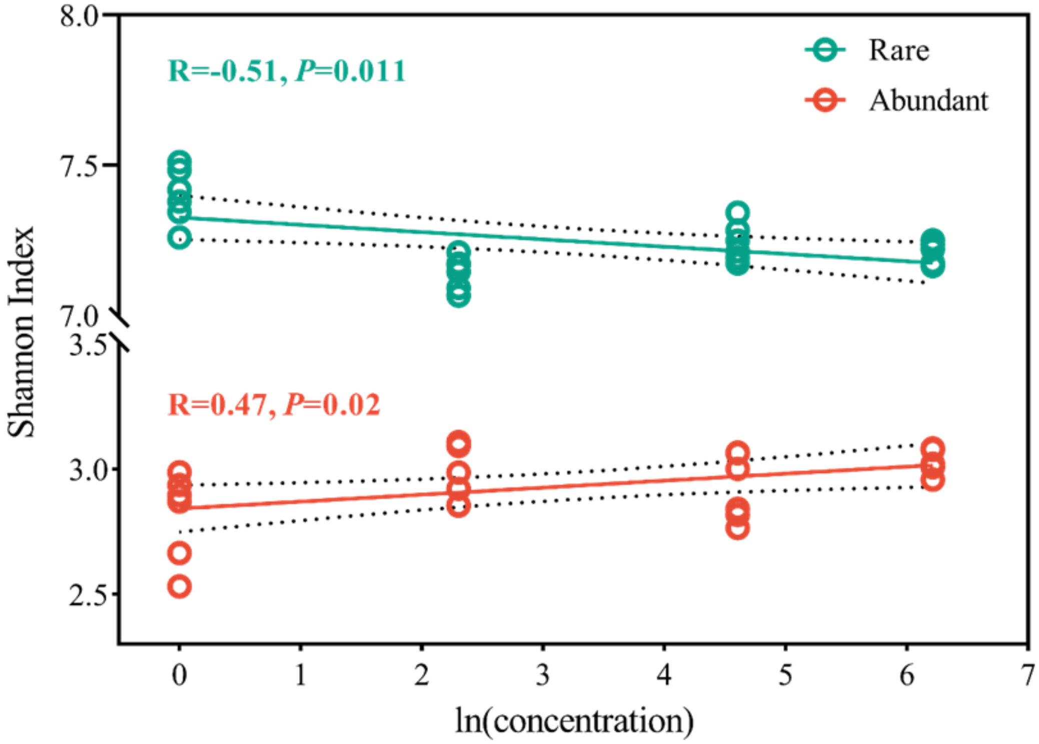

Figure S3 Correlation between natural logarithm of pyrene concentrations and Shannon-Wiener index of abundant and rare bacterial taxa

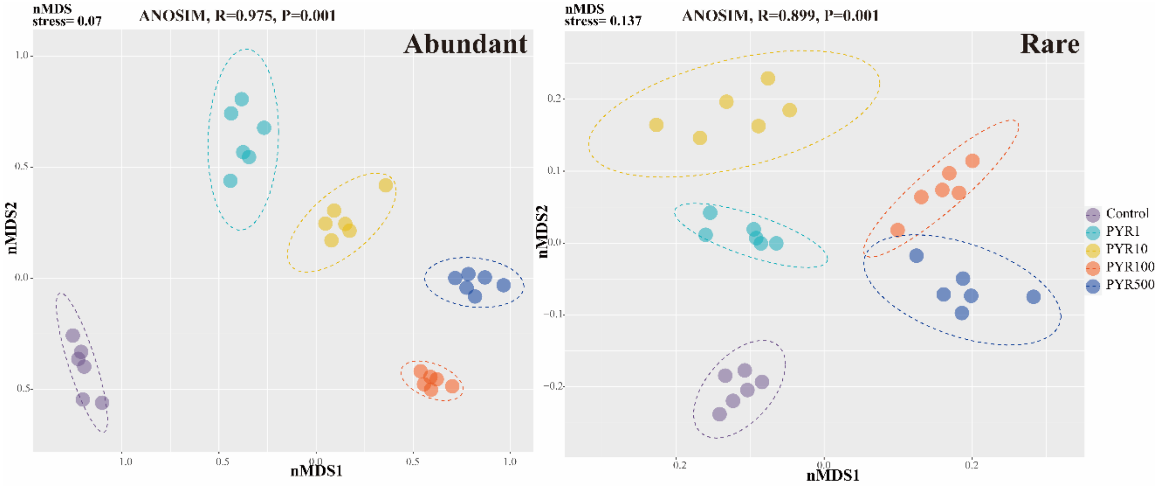

Figure S4 nMDS was calculated based on Bray-Curtis dissimilarity and 95% confidence ellipse was added for each treatment.

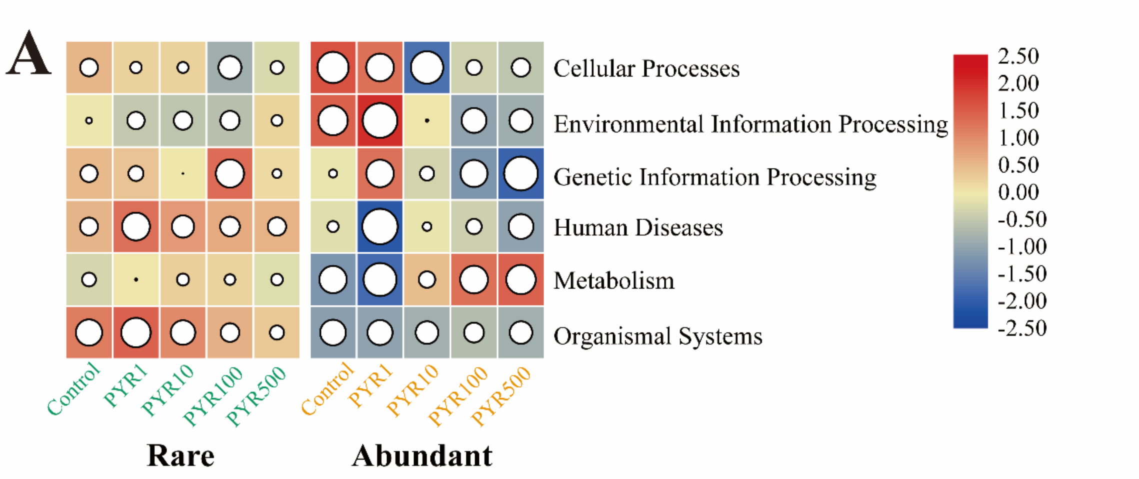

B

Level1	Level2	Level3	ko
Environmental Information Processing	Signaling molecules and interaction	Cytokine-cytokine receptor interaction	ko04060
Cellular Processes	Transport and catabolism	Phagosome	ko04145
Cellular Processes	Cellular community - eukaryotes	Adherens junction	ko04520
Cellular Processes	Cellular community - eukaryotes	Tight junction	ko04530
Organismal Systems	Immune system	Leukocyte transendothelial migration	ko04670

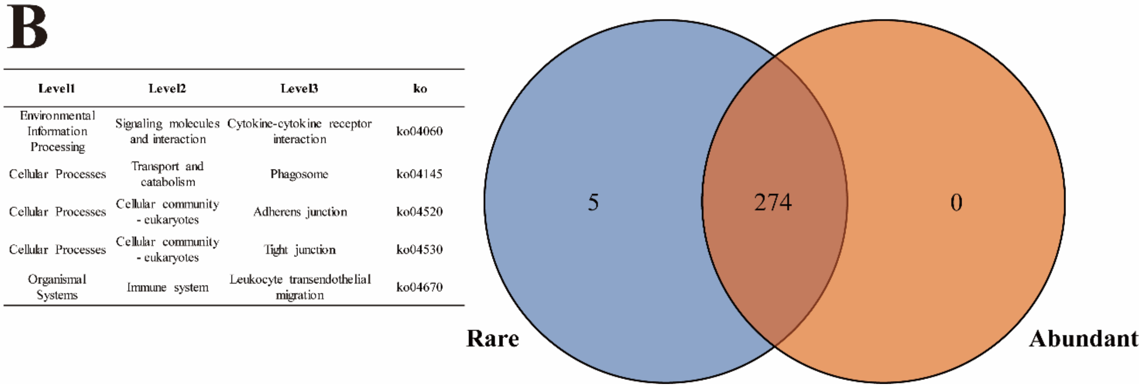

Figure S5 Comparison of potential functions in abundant and rare subcommunities among treatments based on Tax4Fun. (A) Heatmap of KEGG level 2 modules in all five treatments. Color points and circle sizes indicate correlation coefficient; (B) The Venn diagram of the prediction functions of abundant and rare subcommunities.

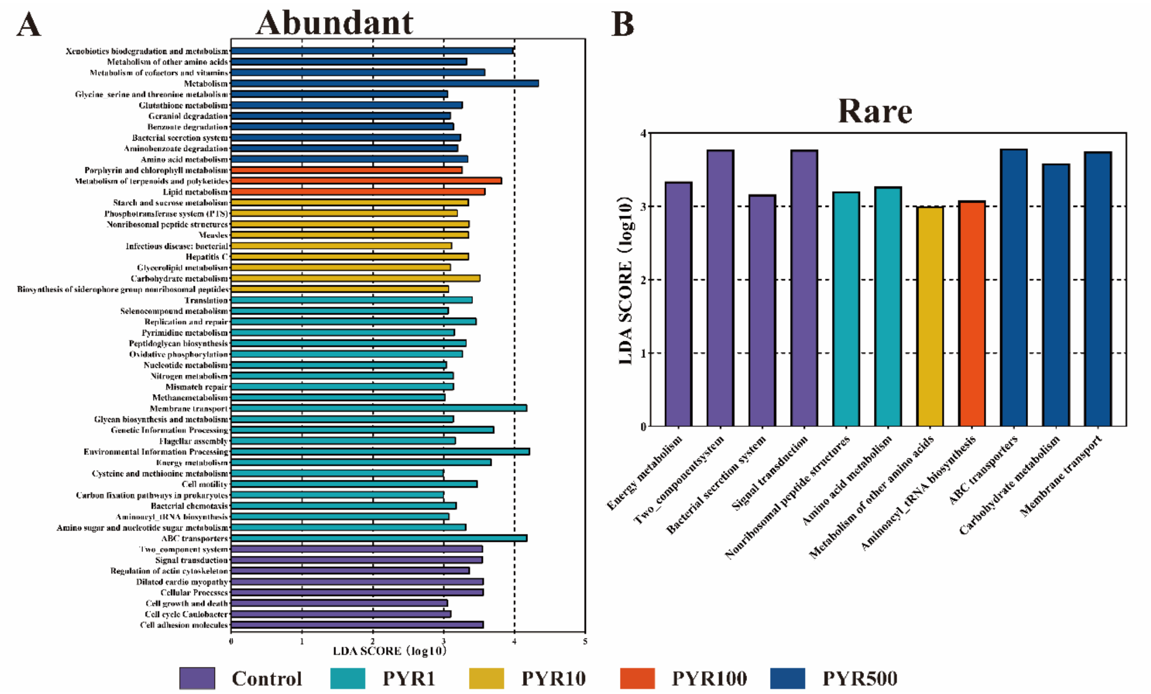

Figure S6 The linear discriminant analysis (LDA) value distribution histogram of functions in abundant (A) and rare (B) subcommunities.

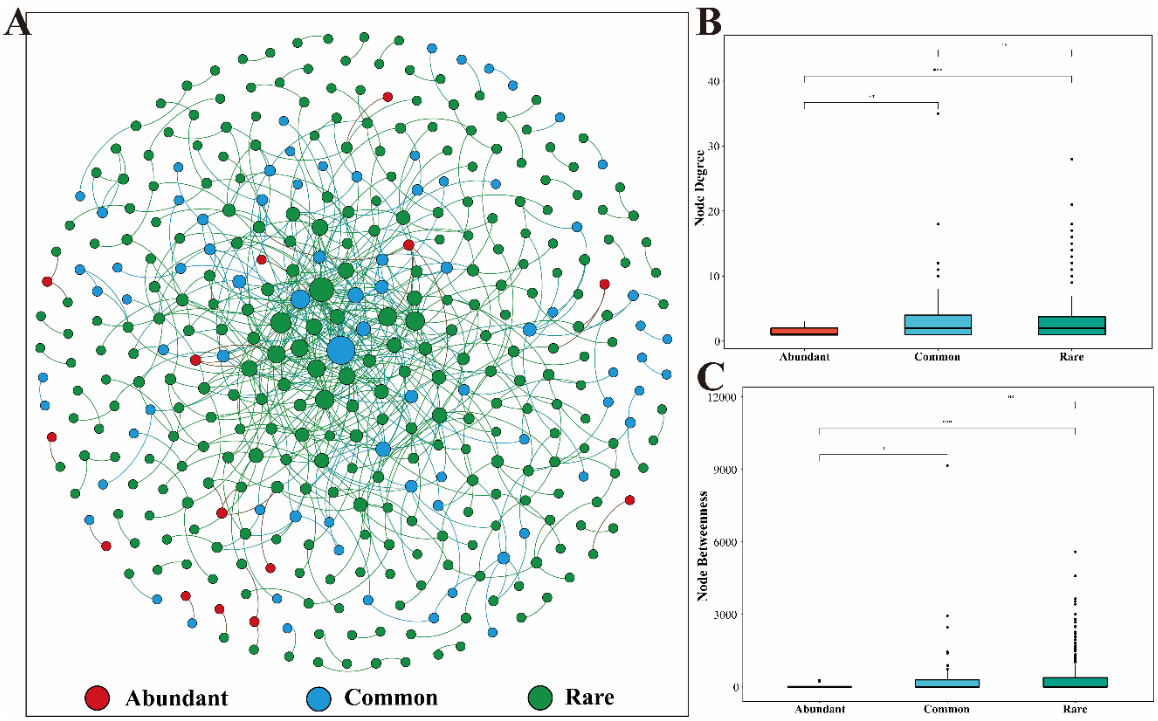

Figure S7 (A) Network in the whole communities. Node degree (B) and betweenness (C) of the network.

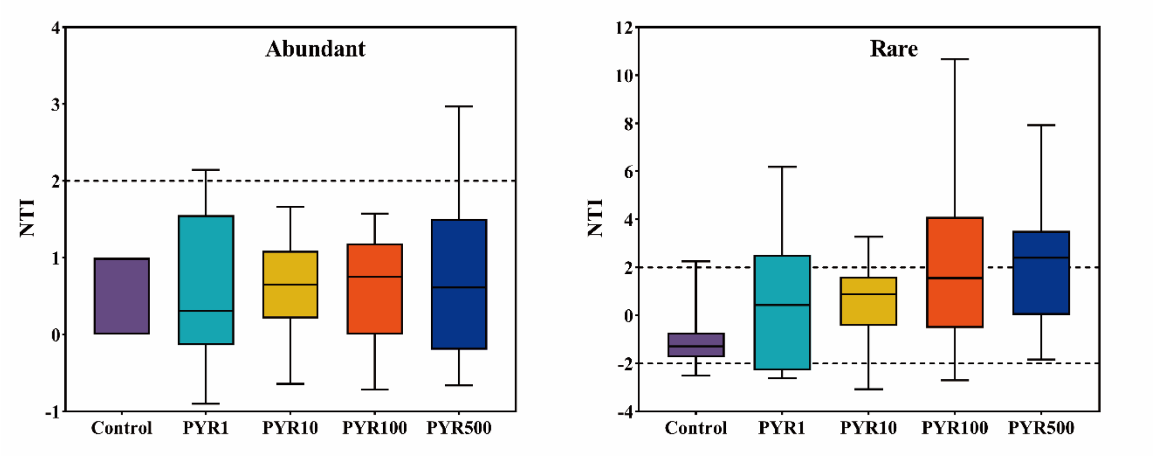

Figure S8 NTI of abundant and rare bacterial taxa under different levels of pyrene stresses based on the null module.

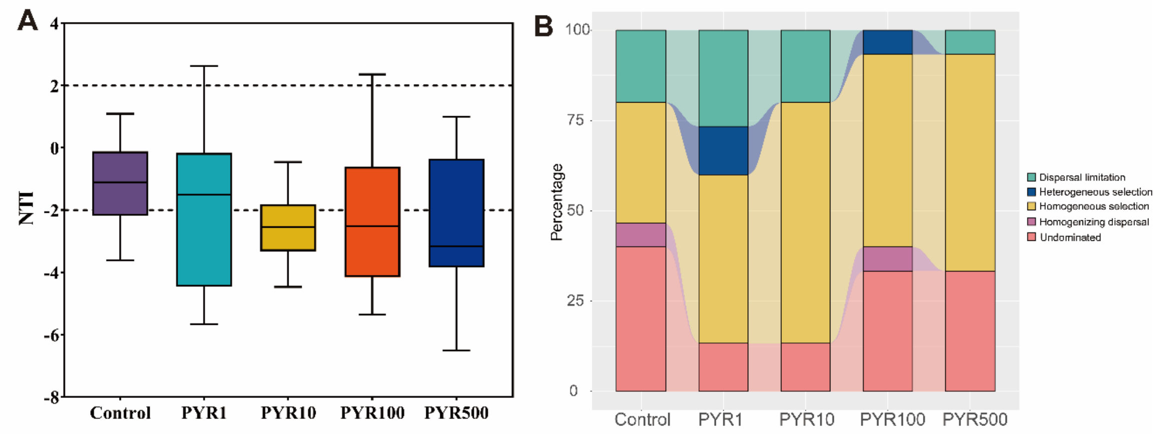

Figure S9 The NTI value (A) and fraction (B) of assembly mechanism in entire community based on the null model.

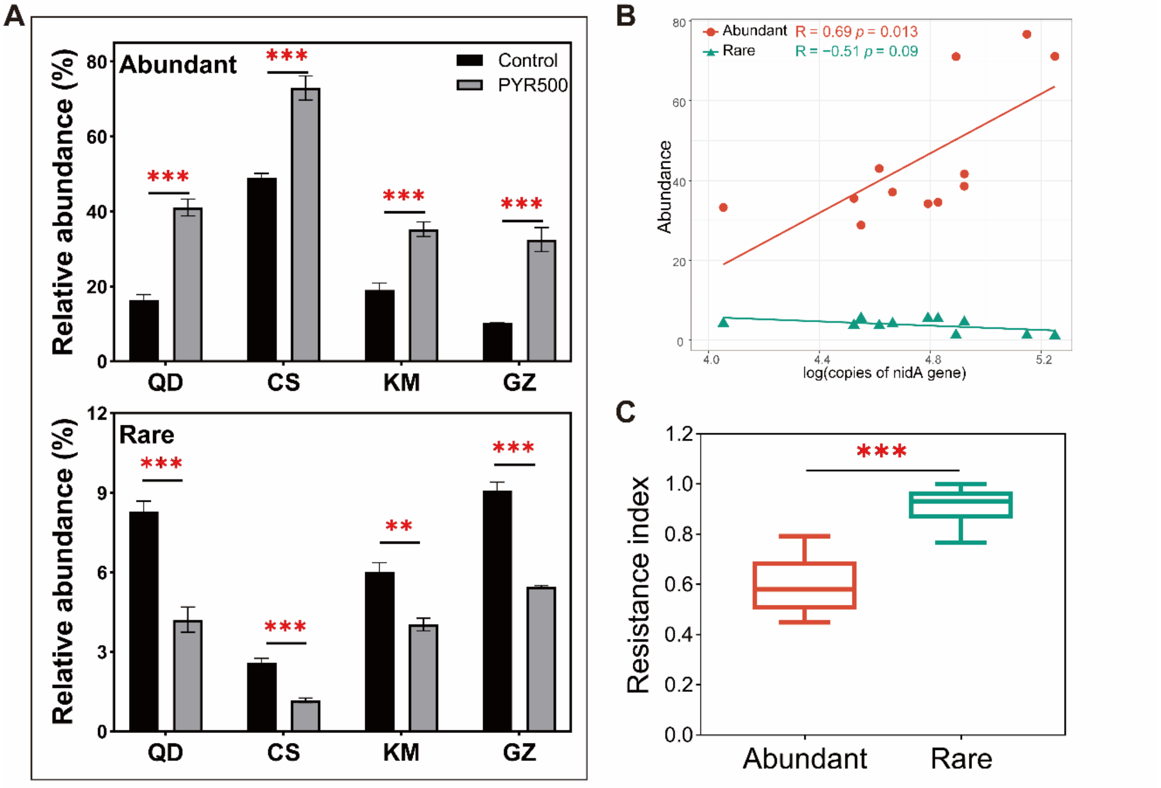

Figure S10 Changes of different soils in abundant and rare taxa under pyrene stresses. (A) the relative abundance of abundant and rare taxa; (B) the correlation analysis between the abundance of abundant and rare taxa and pyrene degradation rate or logarithm of *nidA* gene copies; (C) the resistance index of abundant and rare taxa. Asterisks indicate significance: * $P < 0.05$, ** $P < 0.01$, *** $P<0.001$ based on Tukey's HSD test.

Table S1 Information of abundant OTUs in treatments

OTU	Control	PYR1	PYR10	PYR100	PYR500	Phylum	Class	Order	Family	Genus
OTU_3709	Abundant	Abundant	Abundant	Abundant	Abundant	Firmicutes	Bacilli	Bacillales	Bacillaceae	Bacillus
OTU_4	Abundant	Abundant	Abundant	Abundant	Abundant	Patescibacteria	WWE3	Candidatus Berkelbacteria bacterium RIFCSPHIGHO2_12_FULL_50_11	Candidatus Berkelbacteria bacterium RIFCSPHIGHO2_12_FULL_50_11	Candidatus Berkelbacteria RIFCSPHIGHO2_12_FULL_50_11
OTU_5	Abundant	Abundant	Abundant	Abundant	Abundant	Proteobacteria	Alphaproteobacteria	Sphingomonadales	Sphingomonadaceae	Sphingomonas
OTU_7	Abundant	Abundant	Abundant	Abundant	Abundant	Proteobacteria	Alphaproteobacteria	Rhodospirillales	Magnetospirillaceae	
OTU_26	Abundant	Common	Abundant	Abundant	Abundant	Firmicutes	Clostridia	Clostridiales	Family XVIII	
OTU_29128	Abundant	Abundant	Abundant	Common	Abundant	Firmicutes	Clostridia	Clostridiales	Family XVIII	
OTU_35147	Abundant	Common	Common	Common	Rare	Proteobacteria	Gammaproteobacteria	Pseudomonadales	Pseudomonadaceae	Pseudomonas
OTU_5111	Abundant	Common	Common	Abundant	Common	Proteobacteria	Gammaproteobacteria	Betaproteobacteriales	Rhodocyclaceae	Azoarcus
OTU_19	Abundant	Abundant	Abundant	Common	Common	Actinobacteria	Actinobacteria	Propionibacteriales	Nocardioidaceae	Aeromicrobium
OTU_38	Abundant	Abundant	Abundant	Common	Common	Proteobacteria	Gammaproteobacteria	Betaproteobacteriales	Rhodocyclaceae	
OTU_69	Abundant	Common	Rare	Common	Common	Bacteroidetes	Bacteroidia	Cytophagales	Microscillaceae	Ohtaekwangia
OTU_6	Abundant	Abundant	Common	Common	Common	Firmicutes	Clostridia	Clostridiales	Family XVIII	
OTU_40024	Abundant	Rare	Common	Common	Common	Firmicutes	Clostridia	Clostridiales	Family XVIII	
OTU_125	Abundant	Common	Common	Common	Common	Patescibacteria	Saccharimonadia	Saccharimonadales	uncultured bacterium	
OTU_175	Abundant	Common	Common	Common	Common	Bacteroidetes	Bacteroidia	Bacteroidetes VC2.1 Bac22	metagenome	
OTU_2801	Abundant	Common	Common	Common	Common	Proteobacteria	Gammaproteobacteria	Betaproteobacteriales	Rhodocyclaceae	Methyloversatilis
OTU_38603	Abundant	Common	Common	Common	Common	Patescibacteria	Saccharimonadia	Saccharimonadales	uncultured bacterium	

OTU_58	Abundant	Common	Common	Common	Common	Bacteroidetes	Bacteroidia	Cytophagales	Microscillaceae	Ohtaekwangia
OTU_43	Rare	Common	Common	Abundant	Abundant	Bacteroidetes	Bacteroidia	Sphingobacteriales	env.OPS 17	
OTU_18376	Rare	Common	Abundant	Common	Abundant	Proteobacteria	Alphaproteobacteria	Rhodospirillales	Magnetospirillaceae	
OTU_297	Rare	Common	Rare	Common	Abundant	Patescibacteria	Parcubacteria	Candidatus Kaiserbacteria	uncultured bacterium	
OTU_1257	Rare	Common	Common	Common	Abundant	Proteobacteria	Alphaproteobacteria	Rhodospirillales	Magnetospirillaceae	Magnetospirillum
OTU_7565	Rare	Common	Abundant	Rare	Common	Bacteroidetes	Bacteroidia	Sphingobacteriales	env.OPS 17	
OTU_53	Rare	Abundant	Abundant	Common	Common	Patescibacteria	Parcubacteria	Candidatus Kaiserbacteria	uncultured bacterium	
OTU_335	Rare	Common	Abundant	Common	Common	Proteobacteria	Deltaproteobacteria	Bdellovibrionales	Bdellovibrionaceae	Bdellovibrio
OTU_157	Rare	Common	Abundant	Common	Common	Bacteroidetes	Bacteroidia	Sphingobacteriales	env.OPS 17	
OTU_2	Common	Abundant	Abundant	Abundant	Abundant	Proteobacteria	Gammaproteobacteria	Betaproteobacteriales	Burkholderiaceae	Ramlibacter
OTU_27546	Common	Abundant	Abundant	Abundant	Abundant	Proteobacteria	Gammaproteobacteria	Betaproteobacteriales	Burkholderiaceae	
OTU_3	Common	Abundant	Abundant	Abundant	Abundant	Firmicutes	Bacilli	Bacillales	Bacillaceae	Bacillus
OTU_8	Common	Abundant	Abundant	Abundant	Abundant	Firmicutes	Bacilli	Bacillales	Paenibacillaceae	Ammoniphilus
OTU_10	Common	Common	Abundant	Abundant	Abundant	Chloroflexi	Anaerolineae	SBR1031	A4b	OLB13
OTU_14	Common	Common	Abundant	Abundant	Abundant	Deinococcus-Thermus	Deinococci	Deinococcales	Deinococcaceae	
OTU_15	Common	Common	Abundant	Abundant	Abundant	Acidobacteria	Subgroup 6	uncultured bacterium	uncultured bacterium	
OTU_50	Common	Common	Abundant	Abundant	Abundant	Actinobacteria	Actinobacteria	Corynebacteriales	Mycobacteriaceae	Mycobacterium
OTU_1	Common	Abundant	Common	Abundant	Abundant	Proteobacteria	Alphaproteobacteria	Rhodospirillales	Magnetospirillaceae	Magnetospirillum
OTU_21	Common	Common	Common	Abundant	Abundant	Proteobacteria	Deltaproteobacteria	Myxococcales	Blfdi19	metagenome
OTU_33	Common	Common	Common	Abundant	Abundant	Proteobacteria	Alphaproteobacteria	Caulobacterales	Caulobacteraceae	Brevundimonas
OTU_52	Common	Common	Common	Abundant	Abundant	Proteobacteria	Gammaproteobacteria	Betaproteobacteriales	Burkholderiaceae	Noviherbaspirillum
OTU_7879	Common	Common	Common	Abundant	Abundant	Proteobacteria	Gammaproteobacteria	Betaproteobacteriales	Burkholderiaceae	

OTU_27	Common	Abundant	Abundant	Common	Abundant	Proteobacteria	Gammaproteobacteria	Betaproteobacteriales	Burkholderiaceae	Noviherbaspirillum
OTU_45	Common	Common	Abundant	Common	Abundant	Firmicutes	Clostridia	Clostridiales	Family XVIII	
OTU_61	Common	Common	Abundant	Common	Abundant	Proteobacteria	Alphaproteobacteria	Rhizobiales	Beijerinckiaceae	Microvirga
OTU_171	Common	Common	Common	Common	Abundant	Patescibacteria	Parcubacteria	Candidatus Kaiserbacteria	uncultured bacterium	
OTU_7348	Common	Common	Common	Common	Abundant	Firmicutes	Clostridia	Clostridiales	Family XVIII	
OTU_107	Common	Common	Common	Common	Abundant	Firmicutes	Clostridia	Clostridiales	Family XVIII	
OTU_10723	Common	Common	Common	Common	Abundant	Firmicutes	Clostridia	Clostridiales	Family XVIII	
OTU_18	Common	Common	Common	Common	Abundant	Bacteroidetes	Bacteroidia	Chitinophagales	Chitinophagaceae	Flavisolibacter
OTU_27041	Common	Common	Common	Common	Abundant	Proteobacteria	Alphaproteobacteria	Sphingomonadales	Sphingomonadaceae	Sphingomonas
OTU_29	Common	Common	Common	Common	Abundant	Actinobacteria	Actinobacteria	Corynebacteriales	Nocardiaceae	Rhodococcus
OTU_35	Common	Common	Common	Common	Abundant	Proteobacteria	Gammaproteobacteria	Betaproteobacteriales	Rhodocyclaceae	Azoarcus
OTU_37	Common	Common	Common	Common	Abundant	Proteobacteria	Alphaproteobacteria	Rhizobiales	Rhizobiales Incertae Sedis	
OTU_4276	Common	Common	Common	Common	Abundant	Proteobacteria	Alphaproteobacteria	Caulobacterales	Caulobacteraceae	Brevundimonas
OTU_47	Common	Common	Common	Common	Abundant	Bacteroidetes	Bacteroidia	Sphingobacteriales	KD3-93	
OTU_120	Common	Abundant	Common	Common	Rare	Patescibacteria	Saccharimonadia	Saccharimonadales	uncultured bacterium	
OTU_57	Common	Common	Abundant	Abundant	Common	Bacteroidetes	Bacteroidia	Unclassified	Unclassified	
OTU_78	Common	Common	Abundant	Abundant	Common	Patescibacteria	Berkelbacteria	metagenome	metagenome	
OTU_108	Common	Common	Rare	Abundant	Common	Proteobacteria	Alphaproteobacteria	Rickettsiales	Mitochondria	Malawimonas jakobiformis
OTU_21176	Common	Rare	Common	Abundant	Common	Firmicutes	Clostridia	Clostridiales	Family XVIII	
OTU_70	Common	Common	Common	Abundant	Common	Cyanobacteria	Melainabacteria	Vampirovibrionales	uncultured bacterium	
OTU_126	Common	Common	Common	Abundant	Common	Actinobacteria	Actinobacteria	Corynebacteriales	Mycobacteriaceae	Mycobacterium
OTU_24844	Common	Common	Common	Abundant	Common	Proteobacteria	Gammaproteobacteria	Betaproteobacteriales	Burkholderiaceae	Cupriavidus
OTU_40	Common	Common	Common	Abundant	Common	Proteobacteria	Gammaproteobacteria	Betaproteobacteriales	Rhodocyclaceae	
OTU_4179	Common	Common	Common	Abundant	Common	Proteobacteria	Alphaproteobacteria	Sphingomonadales	Sphingomonadaceae	Ellin6055

OTU_80	Common	Common	Common	Abundant	Common	Proteobacteria	Deltaproteobacteria	Bdellovibrionales	Bdellovibrionaceae	Bdellovibrio
OTU_9089	Common	Abundant	Common	Rare	Common	Firmicutes	Clostridia	Clostridiales	Family XVIII	
OTU_13	Common	Abundant	Abundant	Common	Common	Patescibacteria	Saccharimonadia	Saccharimonadales	uncultured bacterium	
OTU_17	Common	Abundant	Abundant	Common	Common	Firmicutes	Clostridia	Clostridiales	Family XVIII	
OTU_20	Common	Abundant	Abundant	Common	Common	Proteobacteria	Gammaproteobacteria	Betaproteobacteriales	Burkholderiaceae	Ramlibacter
OTU_9	Common	Abundant	Abundant	Common	Common	Proteobacteria	Alphaproteobacteria	Caulobacterales	Caulobacteraceae	Phenylobacterium
OTU_13934	Common	Common	Abundant	Common	Common	Actinobacteria	Actinobacteria	Propionibacteriales	Nocardioidaceae	Aeromicrobium
OTU_25	Common	Common	Abundant	Common	Common	Firmicutes	Bacilli	Bacillales	Bacillaceae	Fictibacillus
OTU_36828	Common	Common	Abundant	Common	Common	Proteobacteria	Alphaproteobacteria	Caulobacterales	Caulobacteraceae	Phenylobacterium
OTU_93	Common	Common	Abundant	Common	Common	Proteobacteria	Alphaproteobacteria	Sphingomonadales	Sphingomonadaceae	Sphingomonas
OTU_22	Common	Abundant	Rare	Common	Common	Firmicutes	Clostridia	Clostridiales	Family XVIII	
OTU_128	Common	Abundant	Common	Common	Common	Chloroflexi	Anaerolineae	SBR1031	A4b	
OTU_143	Common	Abundant	Common	Common	Common	Unclassified	Unclassified	Unclassified	Unclassified	
OTU_39506	Common	Abundant	Common	Common	Common	Firmicutes	Clostridia	Clostridiales	Family XVIII	
OTU_42	Common	Abundant	Common	Common	Common	Bacteroidetes	Ignavibacteria	OPB56	uncultured bacterium	
OTU_46	Common	Abundant	Common	Common	Common	Patescibacteria	Saccharimonadia	Saccharimonadales	Unclassified	
OTU_5829	Common	Abundant	Common	Common	Common	Proteobacteria	Gammaproteobacteria	Betaproteobacteriales	Burkholderiaceae	
OTU_6300	Common	Abundant	Common	Common	Common	Proteobacteria	Gammaproteobacteria	Betaproteobacteriales	Burkholderiaceae	Herbaspirillum
